# Resistance to 4-(9-acridinylamino) methanesulphon-m-anisidide (m-AMSA) in human myeloid leukaemia.

**DOI:** 10.1038/bjc.1990.11

**Published:** 1990-01

**Authors:** W. L. Skinner, D. Murray, V. Kohli, M. Beran, K. B. McCredie, E. J. Freireich, B. S. Andersson

**Affiliations:** Department of Hematology, University of Texas M.D. Anderson Cancer Center, Houston 77030.

## Abstract

**Images:**


Br. J. Cancer (1990), 61, 51–55 © Macmillan Press Ltd., 1990

### Resistance to 4'-(9-acridinylamino) methanesulphon-*m*-anisidide (*m*-AMSA) in human myeloid leukaemia

W.L. Skinner[1], D. Murray[2], V. Kohli[1], M. Beran[1], K.B. McCredie[1], E. J. Freireich[1] & B.S. Andersson[1]

*Departments of [1]Hematology and [2]Experimental Radiotherapy, The University of Texas M.D. Anderson Cancer Center, 1515 Holcombe Blvd, Houston, Texas 77030, USA.*

**Summary** Sublines of a human myeloid leukaemia cell line, KBM-3, with increasing degrees of resistance to the antileukaemic agent 4'-(9-acridinlylamino) methanesulphon-*m*-anisidide (*m*-AMSA) were evaluated for their response to this drug using a clonogenic assay to measure cell survival and alkaline elution to assess *m*-AMSA induced DNA strand breakage. Polyacrylamide gel electrophoresis was used to map the protein profiles of the various cell lines. The resistant lines were obtained by intermittent exposure of the KBM-3 cells to the highest tolerated concentration of *m*-AMSA so that the culture would be repopulated only by the most resistant subpopulation after each exposure. Two distinct phases were apparent during the development of resistance. During the first 14 months of intermittent exposure to maximally tolerated concentrations of *m*-AMSA, the cells developed low-degree *m*-AMSA resistance (5–7-fold as compared with the parent line, as measured by cell survival). This low-degree resistance was characterised by a somewhat suppressed level of DNA strand breakage and no measurable change in cellular protein levels. Subsequently, a single escalation of the *m*-AMSA retreatment concentration resulted in a cell population that was approximately 100-fold resistant, as assessed by cloning. This rapid phenotypic change temporally coincided with the acquisition of an almost complete refractoriness to *m*-AMSA-induced DNA strand breakage and the loss of a cellular 76 kDa protein. We suggest that the loss of this protein is important for the development of a highly *m*-AMSA resistant phenotype.

The emergence of drug resistance during the treatment of malignant disease continues to be a major reason for therapeutic failure. Understanding the cellular mechanisms for the development of drug resistance is therefore of major importance for the successful circumvention of this problem. Such mechanistic studies are greatly facilitated by the availability of continuously growing tumour cell lines with experimentally induced resistance to different antineoplastic agents.

Human acute myelogenous leukamia (AML) cell lines such as HL-60 (Collins *et al.*, 1977; Gallagher *et al.*, 1979) have been used to generate models of resistance to the antileukaemic agent 4'-(9-acridinylamino) methanesulphon-*m*-anisidide (*m*-AMSA) (Odaimi *et al.*, 1986; Beran & Andersson 1987). Our previous studies of HL-60/AMSA showed that the highly resistant cells had significantly fewer *m*-AMSA induced topoisomerase II mediated DNA strand breaks than the drug sensitive parent line (Bakic *et al.*, 1986). Since it has not been established whether the observed decrease in *m*-AMSA induced DNA strand breakage is a general mechanism for *m*-AMSA resistance in human AML or a phenomenon uniquely associated with the highly resistant HL-60 cell line, we expanded our investigation to a second human AML cell line, KBM-3. It is also questionable whether the phenomena observed in highly resistant cell lines can completely explain the lower degrees of resistance that are likely to be observed in a clinical situation. We therefore examined several sublines of KBM-3 with increasing degrees of resistance to *m*-AMSA. We used the alkaline elution technique to measure DNA strand scission in each of these cell lines after exposure to various concentrations of *m*-AMSA and correlated the results with the phenotypic expression of resistance as measured by clonogenic assay.

In a preliminary study we analysed total cellular proteins from HL-60, KBM-3 and their respective high-degree *m*-AMSA resistant sublines; these initial results suggested the disappearance of a specific protein band with a molecular mass of 76 kDa in the highly resistant sublines, although no temporal relationship between the development of *m*-AMSA resistance and the disappearance of the protein was established (Kohli *et al.*, 1987). In the present study we have examined the time course of the loss of this 76 kDa protein during the acquisition of *m*-AMSA resistance in KBM-3 cells.

Our results indicate that there are two phases in the cellular development of resistance to *m*-AMSA in human AML. The first of these processes results in low-degree resistance and in a slight but measurable decrease in *m*-AMSA induced DNA strand breaks. The subsequent abrupt transition to a highly resistant phenotype is temporally related to a refractoriness to *m*-AMSA induced DNA strand scission and the disappearance of the 76 kDa protein.

### Materials and methods

### *Cell lines*

The KBM-3 cell line was established in 1983 from a patient with AML at The University of Texas M.D. Anderson Cancer Center. The cells have an immature monocytic phenotype and are grown in suspension in Iscove's modification of Dulbecco's minimal essential medium (IMDM) (GIBCO Laboratories, Grand Island, NY, USA) supplemented with 10% heat-inactivated fetal calf serum (FCS) (Irvine Scientific, Santa Ana, CA, USA). Under these conditions the cells have a doubling time of approximately 23 h and a plating efficiency in soft agar of about 15%. The *m*-AMSA resistant sublines were derived over a period of 18 months through intermittent 60 min exposures at 37°C to gradually escalating concentrations of the drug added to the suspension cultures. The cells were treated three times at each concentration level, each treatment being 1–2 weeks apart. The exact treatment protocol is shown in Figure 1. The degree of escalation of *m*-AMSA concentration and the time interval between treatments was chosen such that only a few cells (i.e. the most resistant cells in the population) would survive the treatment and subsequently repopulate the culture. This protocol should therefore result in the most rapidly achievable development of resistance and it approximates the clinical situation where DNA-intercalating agents such as *m*-AMSA are used in intermittent high-dose chemotherapy. The cellular drug tolerance increased slowly over the first 440

Correspondence: B.S. Andersson.
Received 4 May 1989; and in revised form 11 August 1989.

**Figure 1** Protocol used for the development of m-AMSA resistant sublines of KBM-3; the cells were intermittently exposed to m-AMSA in gradually escalating concentrations for 60 min at 37°C. The points marked on this curve represent only the particular sublines that were studied in the present investigation: (●) KBM-3; (▽) KBM-3/AMSA 6.25; (◆) KBM-3/AMSA 10; (○) KBM-3/AMSA 12.5; (▼) KBM-3/AMSA 15; (□) KBM-3/AMSA 20.

days and then rapidly over a relatively short period of time (Figure 1).

Before escalation of the m-AMSA concentration, some cells were preserved in liquid nitrogen, their designation being that of the tolerated drug concentration at the time of cryopreservation. These preserved cells also served as backups against losing the cell line since the escalated retreatment concentration of m-AMSA was specifically chosen to be close to the limit of tolerance, and indeed on rare occasions no surviving cells could be recovered.

The cells used in this study were designated as KBM-3/AMSA 6.25, KBM-3/AMSA 10, KBM-3/AMSA 12.5, KBM-3/AMSA 15, and KBM-3/AMSA 20. The numerical suffix refers to the drug concentration in μM units (60 min treatment at 37°C) that was necessary to inhibit appreciably the proliferation of the cells in suspension at their particular level of acquired resistance. This value therefore provides a very crude estimate of the level of resistance; a more precise estimate of resistance was determined from the survival of the cells in a clonogenic assay, as described below. In preparation for cloning and elution experiments, the cells were thawed and passaged twice for 7–10 days in IMDM with 10% FCS.

### *m-AMSA treatment conditions*

Leukaemic cells in early log-phase at a density of $10^6$ cells $ml^{-1}$ were exposed for 1 h at 37°C to a series of dilutions of m-AMSA in phosphate-buffered saline (PBS), pH 7.2, enriched with glucose, calcium, magnesium and 5% FCS. Cells incubated in drug-free enriched PBS served as controls. After incubation, the cells were washed twice in ice-cold PBS followed by a 10 min centrifugation at 200 **g**. They were then resuspended in IMDM and used for *in vitro* cloning and alkaline elution studies.

### *In vitro cloning*

Control and m-AMSA treated cells were plated at a density of $5 \times 10^3$ cells $ml^{-1}$ and cultured in 35 mm Petri dishes in IMDM supplemented with 20% FCS and 0.3% agar as viscous support. After incubation for 8 days at 37°C in a humidified atmosphere of 5% $CO_2$ 12% $O_2$ balanced with nitrogen to 100%, colonies (clones with more than 50 cells) were counted using an inverted phase contrast microscope. The surviving fraction at each drug concentration was calculated by comparing the number of colonies growing after drug exposure to the colony growth of cells incubated in drug-free enriched PBS. Survival curves were constructed, $IC_{50}$ (the drug concentration that inhibits 50% of colony formation) values were calculated, and the resistance index was determined; the resistance index was defined as the $IC_{50}$ of the resistant line divided by the $IC_{50}$ of the parent KBM-3 cell line.

### *Alkaline elution*

DNA strand breaks were estimated using the alkaline elution methodology developed by Kohn and co-workers (Kohn, 1979, Kohn *et al.*, 1981) with some modifications. Briefly, cells in early exponential growth phase were labelled overnight with methyl $^{14}C$-thymidine (25 nCi $ml^{-1}$) (Amersham International, Amersham, UK) and then chased for 6 h in IMDM supplemented with 10% FCS. After m-AMSA treatment, $9 \times 10^5$ cells were gently deposited on to a 25 mm diameter 2 μm pore polycarbonate membrane (Nuclepore, Pleasanton, CA, USA) and then rinsed twice with ice-cold PBS containing 5 mM EDTA. The cells were lysed with 10 ml of sodium dodecyl sulphate (SDS) (Fisher Scientific, Fair Lawn, NJ, USA) lysis solution (25 mM $Na_4$ EDTA/2% SDS, pH 9.7) containing proteinase K (0.5 mg $ml^{-1}$) (EM Reagents, Darmstadt, FRG). Proteinase K was included in the lysis solution in all of these studies since it has previously been demonstrated that all strand breaks in the KBM-3 and HL-60 cell lines are protein associated (Bakic *et al.*, 1986). The lysis solution was retained in contact with the sample for 30 min at room temperature. The cell lysates were then rinsed twice with 5 ml of 20 mM $Na_4$ EDTA (pH 10.3), and the DNA was eluted overnight in the dark with 0.1 M tetrapropylammonium hydroxide (10% aqueous solution, RSA Chemical Co., Ardsley, NY,. USA)/20 mM $H_4$EDTA/0.1% SDS (w/v) (pH 12.15) at a constant flow rate of 0.04 ml $min^{-1}$ to give 10 equal fractions of approximately 3.5 ml. The DNA in the resulting fractions and that recovered from the interior of the membrane holder after vigorous flushing with 3 ml of 0.4 M NaOH were measured by liquid scintillation counting using Liquiscint scintillation fluid (National Diagnostics, Manville, NJ, USA) in a Tri Carb 4530 scintillation counter (Packard Instruments, Laguna Hills, CA, USA). Any DNA remaining on the membranes was recovered by heating for 1 h at 60°C in 1 M HCl (0.4 ml) followed by the addition of 0.4 M NaOH (2.5 ml) for 30 min at room temperature and was again assayed by liquid scintillation counting.

Strand scission factors (SSFs) were calculated from the resulting elution profiles as the absolute value of the log of the ratio of the percentage of DNA retained on the membrane for the m-AMSA treated sample (after an eluted volume of 21.0 ml) to the percentage of DNA retained for the untreated control sample (again after an eluted volume of 21.0 ml). These SSF values were then expressed as Gray-equivalents of X-ray induced DNA single-strand breaks by using a calibration curve for SSF versus X-ray dose; this calibration curve was derived from an accumulation of data obtained for KBM-3 parent cells that were irradiated on ice with 250 kVp X-rays (General Electric Maximar Unit, dose rate 3.3 Gy $min^{-1}$) and analysed for SSB induction by alkaline elution on the same day as the experiments with m-AMSA-treated cells. All data are the average of at least three separate experiments.

### *Protein analysis*

Exponentially growing cells were collected by centrifugation, washed with PBS, and lysed in 0.5% Nonidet P-40/0.25 M sucrose in 10 mM Tris/HCl (pH 7.2) buffer containing protease inhibitors (10 mM sodium fluoride, 0.5 mM phenylmethylsulphonyl fluoride, 5 μg $ml^{-1}$ leupeptin, 5 μg $ml^{-1}$ aprotinin, 1 mM N-ethyl-maleimide, and 0.25 mM p-hydroxymercuribenzoate) (all from Sigma Chemical, St Louis, MO, USA). The Nonidet P-40 lysates were centrifuged at 14,000 g for 1 min in an Eppendorff microcentrifuge. The supernatant was subsequently subjected to polyarcrylamide gel electrophoresis (PAGE) (Laemmli, 1970;

O'Farrel & O'Farrel, 1977), using a Bio-Rad Protean II gel electrophoresis apparatus (Bio-Rad Inc., Richmond, CA, USA). Proteins were visualised by staining with Coomassie brilliant blue, followed by destaining.

### Results

The resistant phenotypes in the KBM-3 cell lines were developed through intermittent exposure to maximally tolerated concentrations of *m*-AMSA. Sublines from the specific time-points indicated on the curve in Figure 1 were analysed for their response to *m*-AMSA by alkaline elution and cloning and for their protein content by PAGE.

The results of the survival studies on KBM-3 and its resistant sublines after a 60 min exposure to various concentrations of *m*-AMSA are shown in Figure 2. The colony forming ability of all cell lines decreased progressively upon exposure to increasing concentrations of *m*-AMSA. The $IC_{50}$ values showed a 7-fold increase from the parent KBM-3 line up to the KBM-3/AMSA 10 subline, at which point a pronounced shift was observed, reflected by the dramatic increase in the resistance index for the KBM-3/AMSA 12.5, KBM-3/AMSA 15, and KBM-3/AMSA 20 sublines (Table I). In fact, the marked increase in resistance index between KBM-3/AMSA 10 and KBM-3/AMSA 12.5 occurred following a *single* escalation of the *m*-AMSA retreatment concentration.

We used the sensitive alkaline elution method to compare the level of DNA strand break induction in the KBM-3 cell line and its *m*-AMSA-resistant sublines, since previous studies had shown that the highly resistant HL-60/AMSA phenotype exhibited a markedly reduced level of *m*-AMSA induced DNA strand breakage (Bakic *et al.*, 1986). The compiled dose-response curves for the parent KBM-3 line and for four sublines with increasing degrees of resistance to *m*-AMSA are displayed in Figure 3. The two low-degree resistance sublines, KBM-3/AMSA 6.25 and KBM-3/AMSA 10, had slightly depressed levels of DNA strand breaks compared with the parent line. In these two sublines, and in the parent line, the maximum level of DNA breakage was achieved with an *m*-AMSA concentration of about 0.5 μM. A dramatic change in DNA breakage frequency was observed between the KBM-3/AMSA 10 and KBM-3/AMSA 12.5 sublines; the highly resistant phenotype, exemplified by KBM-3 AMSA 12.5, KBM-3/AMSA 15 and KBM-3/AMSA 20, manifested a complete absence of *m*-AMSA induced DNA strand breaks over this same range of drug concentration (i.e.

**Figure 2** *In vitro* survival of sensitive KBM-3 cells, and KBM-3 sublines with increasing *m*-AMSA resistance, as a function of the concentration of *m*-AMSA (60 min incubation at 37°C): (●) KBM-3; (▽) KBM-3/AMSA 6.25; (◆) KBM-3/AMSA 10; (○) KBM-3/AMSA 12.5; (▼) KBM-3/AMSA 15; (□) KBM-3/ASMA 20. Points, mean of two independent experiments in which the cells at each drug concentration were plated in triplicate. Bars, s.d.

**Figure 3** Effect of increasing concentrations of *m*-AMSA on the level of protein-associated DNA strand breaks detected in KBM-3 sublines with varying resistance to the drug: (●) KBM-3; (▽) KBM-3/AMSA 6.25; (◆) KBM-3/AMSA 10; (○) KBM-3/AMSA 12.5; (▼) KBM-3/AMSA 15. KBM-3/AMSA 20 cells were identical to KBM-3/AMSA 15 but are not shown for clarity. The drug exposures were for 60 min at 37°C in all cases. Points, mean of three independent experiments. Bars, s.e.

up to 0.5 μM). When the drug concentrations were raised to the same range as the $IC_{50}$ values for these high-degree resistance lines (i.e. up to 50 μM), some concentration-dependent strand breakage was observed, but this effect was much less pronounced than for the parent or low-degree resistance lines exposed to 0.5 μM drug (i.e. concentrations $\leqslant$ their $IC_{50}$).

Figure 4 shows a representative set of the elution profiles used to generate the data in Figure 3. The elution profiles of the KBM-3/AMSA 10 subline and of the KBM-3/AMSA 12.5 subline (which was developed from KBM-3/AMSA 10 cells following a single escalation of the *m*-AMSA retreatment concentration) clearly illustrate the pronounced transition in susceptibility to the DNA-damaging effects of *m*-AMSA that occurred between these particular degrees of resistance.

The results of PAGE analysis for cytoplasmic protein content are shown in Figure 5. The most obvious change was the loss of a 76 kDa protein band, which disappeared from the cells at the same point at which high-degree resistance was observed with alkaline elution and cloning, i.e. at the transition from the KBM-3/AMSA 10 to the KBM-3/AMSA 12.5 subline. Other minor alterations in the protein profiles could be discerned, although these changes were somewhat variable and were impossible to quantitate in a one-dimensional system.

### Discussion

The introduction of *m*-AMSA into clinical trials in 1978 provided a therapeutic alternative for anthracycline-resistant AML, producing complete remissions in about 25–30% of the patients (Legha *et al.*, 1980, 1982). In addition, *m*-AMSA in combination with 1-*B*-D-arabinofuranosylcytosine (Ara-C) produced complete remissions in 70–75% of previously untreated patients with AML (Keating *et al.*, 1987). Unfortunately, these figures have not been translated into a substantial prolongation of remissions or an increased cure rate. When the leukaemia recurs, drug resistance continues to be a major clinical obstacle.

Studies of the mechanism of *m*-AMSA cytotoxicity have

**Figure 4** Typical alkaline elution profiles for KBM-3/AMSA 10 (**a**, ◆) and KBM-3/AMSA 12.5 (**b**, O) cells after exposure to various concentrations of *m*-AMSA for 60 min at 37°C.

**Figure 5** SDS/polyacrylamide gel electrophoresis patterns of cellular proteins extracted from KBM-3, KBM-3/AMSA 6.25, KBM-3/AMSA 10, KBM-3/AMSA 12.5 and KBM-3/AMSA 15. The 76 kDa protein is marked with an arrow. The pattern for KBM-3/AMSA 20 was identical to that for KBM-3/AMSA 15, but has been omitted for clarity.

**Table I** $IC_{50}$ values and resistance indices of KBM-3 cell lines with various degrees of resistance to *m*-AMSA

| *Cell line* | *$IC_{50}$ value (μM)* | *Resistance index*[a] |
|---|---|---|
| KBM-3 | 0.4 | 1.0 |
| KBM-3/AMSA 6.25 | 1.8 | 4.5 |
| KBM-3/AMSA 10 | 2.8 | 7.0 |
| KBM-3/AMSA 12.5 | 38 | 95 |
| KBM-3/AMSA 15 | 40 | 100 |
| KBM-3/AMSA 20 | 45 | 112 |

[a]Defined as $IC_{50}$ of resistant line ÷ $IC_{50}$ of KBM-3.

focused on the role of the interaction between the drug and DNA topoisomerase II in the production of *m*-AMSA induced DNA strand breaks. Such studies have indicated an altered interaction between *m*-AMSA and DNA topoisomerase II in human *m*-AMSA resistant leukaemia (Bakic *et al.*, 1986; Estey *et al.*, 1987). Such mechanistic studies, however, have all been carried out with highly resistant leukaemic cells.

The current investigation used a clonogenic assay, alkaline elution and PAGE to study potentially more clinically relevant sublines of KBM-3 with various degrees of *m*-AMSA resistance. Our data demonstrate two distinct phases in the development of resistance to *m*-AMSA in this human AML line. The first of these phases, corresponding to the first 440 days of intermittent exposure to maximally tolerated concentrations of *m*-AMSA, was characterised by up to 7-fold increase in the resistance index and a small but measurable decrease in the efficiency of DNA strand breakage by *m*-AMSA (Figures 2 and 3, Table I). The second phase, characterised by a dramatic increase in resistance index and a virtual absence of detectable DNA strand breakage after exposure of the cells to the same range of test *m*-AMSA concentrations that produced a high level of strand breakage in the parent KBM-3 line, occurred between the KBM-3/AMSA 10 and the KBM-3/AMSA 12.5 sublines over a short period of time (between days 440 and 480) (Figures 1 and 4). The magnitude of *m*-AMSA induced DNA strand breakage observed using alkaline elution therefore qualitatively paralleled the resistance index obtained from the cloning data.

Because the design of the experimental protocol for the development of *m*-AMSA resistance will result in the elimination of all sensitive cells from the culture (as a result of the extremely cytotoxic concentrations of *m*-AMSA used), the cells within any given population (e.g. KBM-3/AMSA 10) appear to have a relatively uniform resistance to the drug, as indicated by the observations that (i) the elution profiles for highly damaged DNA from such cells (e.g. Figure 4) were essentially linear even after more than 95% of the DNA had been eluted, with no evidence of a resistant tail, and (ii) the survival curves for lines of low-degree resistance also showed no evidence of a resistant tail even at surviving fractions ⩽ 1% (Figure 2).

PAGE analysis of the KBM-3/AMSA cell lines with increasing degrees of resistance revealed the sudden disappearance of a 76 kDa protein band between the KBM-3/AMSA 10 and KBM-3/AMSA 12.5 sublines (Figure 5), i.e. precisely where the transition from low to high-degree resistance occurred. The temporal correlation between the development of the high-degree resistance phenotype and the altered levels of this protein suggests that these two events may be related.

There are several possible explanations for these observations. First, the early development of low-degree resistance may represent either a gradual cellular adaptation to the stress of intermittent drug exposure or a single event occurring at some time during this period. It is not possible at present to discriminate between these two alternatives, although the fact that the maximally tolerated *m*-AMSA concentration did increase progressively with time (Figure 1) favours the former explanation. The more rapid late phase, during which the high-degree resistance phenotype developed concomitantly with a decrease in *m*-AMSA induced DNA single-strand breaks and the disappearance of the 76 kDa protein, may be a result of a separate genetic mutation. Alternatively, the observed two phases in the development of *m*-AMSA resistance may have a common underlying mutational event. The observed development of high-degree resistance at approximately 440 days could result from overexpression or amplification of this mutated gene, causing a suppression of the 76 kDa protein. In this case, both low and high-degree *m*-AMSA resistance would be stable phenotypic changes. The level of the 76 kDa protein may indeed already be decreased in the cells with low-degree *m*-AMSA resistance. However, the sensitivity of our PAGE procedure did not allow the resolution of possible minor changes in 76 kDa protein levels; only the major change concomitant with the cellular transition to a high-degree resistance phenotype could be detected with certainty.

The underlying mechanism(s) of the observed phenomena are therefore at present unknown. That a mutational event is involved at some point is supported by the observation that the HL-60/AMSA (Beran & Andersson, 1987) and KBM-3/AMSA high-degree resistance phenotypes (unpublished data) are extremely stable, with little or no reversion to an *m*-AMSA sensitive phenotype on prolonged culturing without further exposure to the drug. The data suggest a strong temporal correlation between the development of a stable high-degree resistance phenotype, acquisition of refractoriness to *m*-AMSA induced DNA-strand breakage, and the loss of the 76 kDa protein, implying a possible role for this protein in the cytotoxic activity of *m*-AMSA. Studies are under way in our laboratory on the nature and function(s) of the protein and the (genetic ?) event(s) underlying its disappearance from leukaemia cells that become highly *m*-AMSA resistant. The possibility of separate mechanisms for high-degree as opposed to low-degree resistance to the drug, which may be clinically more relevant, is being considered. Studies with clonal populations – e.g. an examination of strand breakage and 76 kDa protein levels in subclones of the KBM-3/AMSA 10 subline – will ultimately clarify many of these questions, as will studies of the stability of the resistance in each of these sublines and their subclones. Clearly, a complete definition of the mechanisms of *m*-AMSA resistance in AML cells await the outcome of such studies.

This work was supported in part by a grant from the Adler Foundation in New York, and by grants CA-28153 and CA-39809 from the National Cancer Institute. Dr Skinner was supported by a Regular Clinical Fellowship position from the American Cancer Society. We wish to thank Ms Dean Anthony for her secretarial assistance in typing this manuscript.

### References

BAKIC, M., BERAN, M., ANDERSSON, B., SILBERMAN, L., ESTEY, E. & ZWELLING, L.A. (1986). Production of topoisomerase II-mediated DNA cleavage in human leukaemia cells predicts their susceptibility to 4'-(9-acridinylamino) methanesulfon-*m*-anisidide (*m*-AMSA). *Biochem. Biophys. Res. Commun.*, **134**, 638.

BERAN, M. & ANDERSSON, B.S. (1987). Development and characterization of a human myelogenous leukemia cell line resistant to 4'-(9-acridinylamino)-3-methanesulfon-*m*-anisidide. *Cancer Res.*, **47**, 1897.

COLLINS, S.J., GALLO, R.C. & GALLAGHER, R.E. (1977). Continuous growth and differentiation of human myeloid leukaemic cells in suspension culture. *Nature*, **270**, 347.

ESTEY, E.H., SILBERMAN, L., BERAN, M., ANDERSSON, B.S. & ZWELLING, L.A. (1987). The interaction between nuclear topoisomerase II activity from human leukemia cells, exogenous DNA, and 4'-(9-acridinylamino) methanesulfon-*m*-anisidide (*m*-AMSA) or 4' (4,6-O-ethylidene-*B*-D-glucopyranoside) (VP-16) indicates the sensitivity of the cells to the drugs. *Biochem. Biophys. Res. Commun.*, **144**, 787.

GALLAGHER, R., COLLINS, S., TRUJILLO, J. & 8 others (1979). Characterisation of the continuous, differentiating myeloid cell line (HL-60) from a patient with acute promyelocytic leukemia. *Blood*, **54**, 713.

KEATING, M.J., GEHAN, E.A., SMITH, T.L. & 5 others (1987). A strategy for evaluation of new treatments in untreated patients: application to a clinical trial of AMSA for acute leukemia. *J. Clin. Oncol.*, **5**, 710.

KOHLI, V., ANDERSSON, B., STECK, P., FREIREICH, E.J. & BERAN, M. (1987). Changes in cellular protein patterns concomitant with acquisition of resistance to a cytotoxic drug *m*-AMSA in two human myelogenous leukemia cell lines HL-60 and KBM-3. *Proc. Am. Assoc. Cancer Res.*, **28**, 288.

KOHN, K.W. (1979). DNA as a target in cancer chemotherapy: measurement of macromolecular DNA damage produced in mammalian cells by anticancer agents and carcinogens. *Methods Cancer Res.*, **16**, 291.

KOHN, K.W., EWIG, R.A.G., ERICKSON, L.C. & ZWELLING, L.A. (1981). Measurement of strand breaks and cross-links by alkaline elution. In *DNA Repair: a Laboratory Manual of Research Procedures*, Friedberg, E.C. & Hanawalt, P.C. (eds) p. 379. Marcel Dekker: New York.

LAEMMLI, U.K. (1970). Cleavage of structural proteins during the assembly of the head of bacteriophage T4. *Nature*, **227**, 680.

LEGHA, S.S., KEATING, M.J., MCCREDIE, K.B., BODEY, G.P. & FREIREICH, E.J. (1982). Evaluation of AMSA in previously treated patients with acute leukemia: results of therapy in 109 adults. *Blood*, **60**, 484.

LEGHA, S.S., KEATING, M.J., ZANDER, A.R., MCCREDIE, K.B., BODEY, G.P. & FREIREICH, E.J. (1980). 4'-(9-acridinylamino) methanesulfon-*m*-anisidide (AMSA): a new drug effective in the treatment of adult acute leukemia. *Ann. Intern. Med.*, **93**, 17.

ODAIMI, M., ANDERSSON, B.S., MCCREDIE, K.B. & BERAN, M. (1986). Drug sensitivity and cross-resistance of the 4'-(9-acridinylamino) methanesulfon-*m*-anisidide-resistant subline of HL-60 human leukemia. *Cancer Res.*, **46**, 3330.

O'FARREL, P.H. & O'FARREL, P.Z. (1977). Two-dimensional polyacrylamide gel electrophoretic fractionation. *Methods Cell Biol.*, **16**, 407.


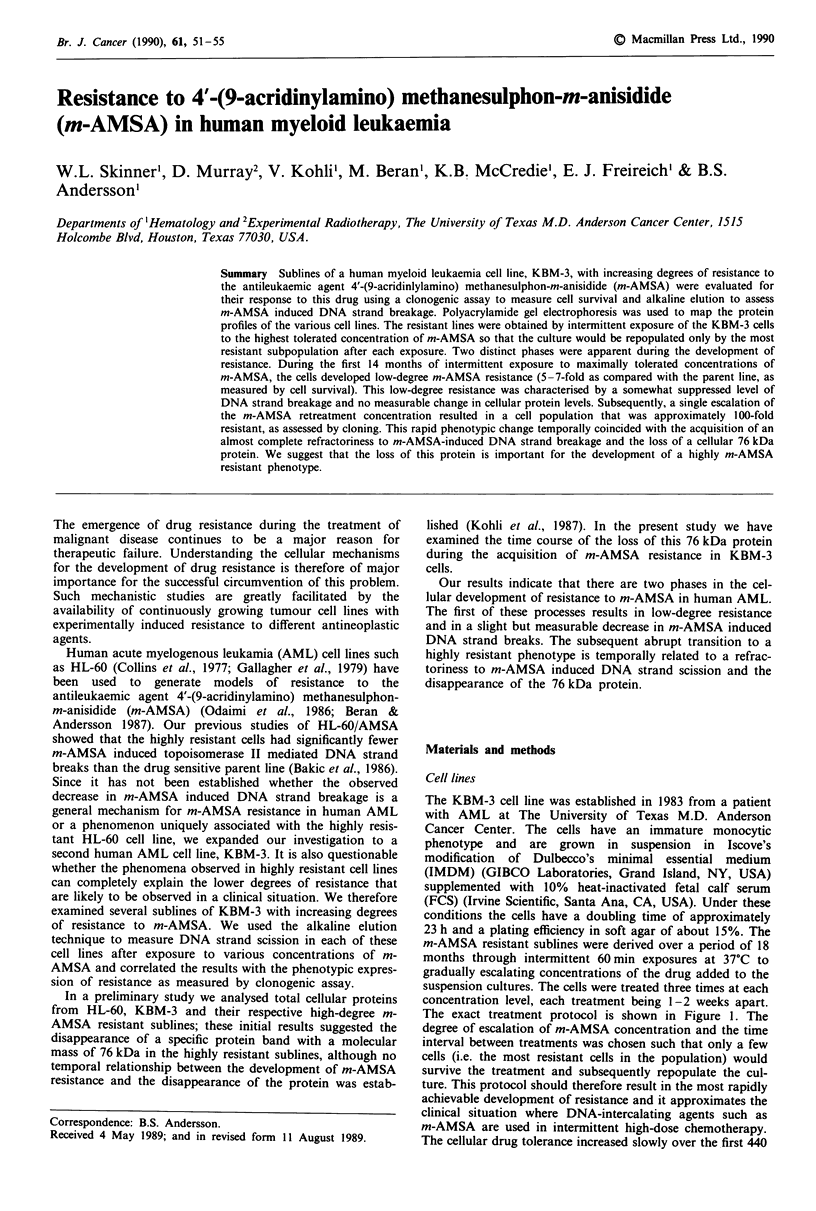

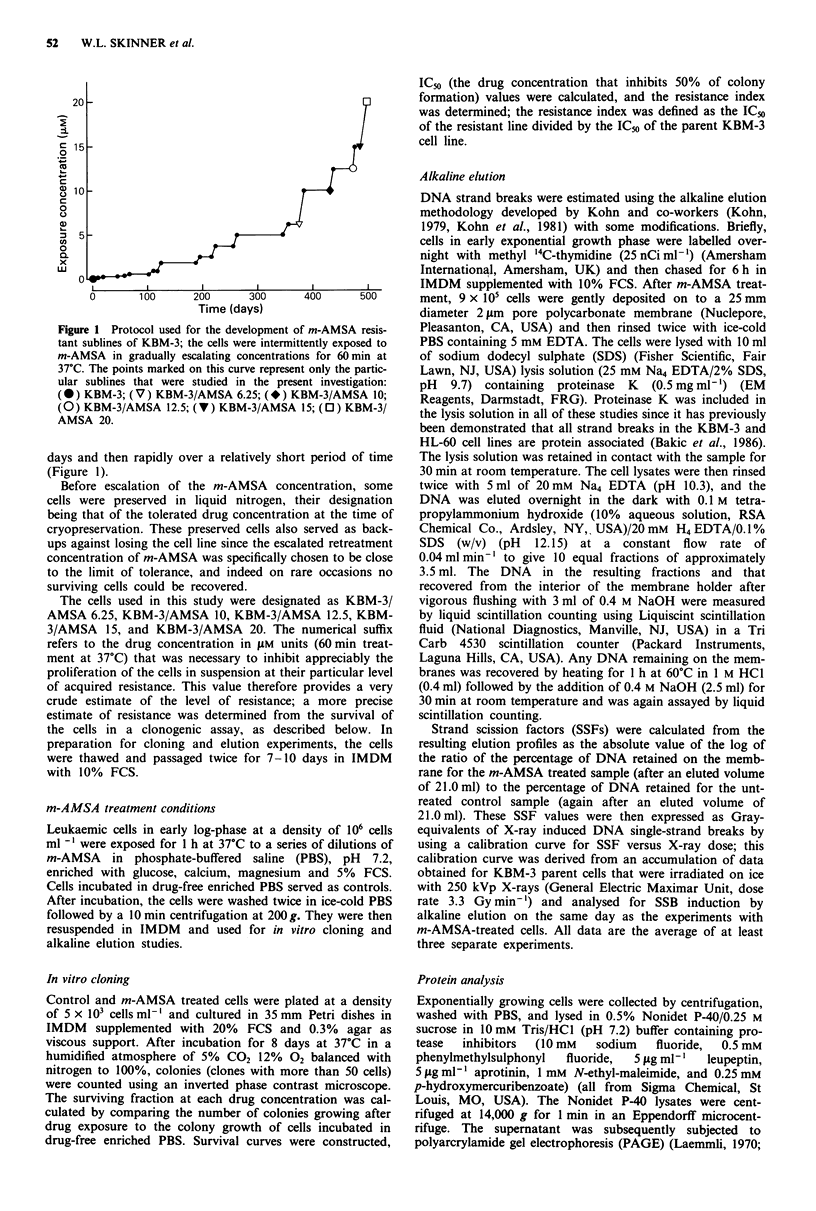

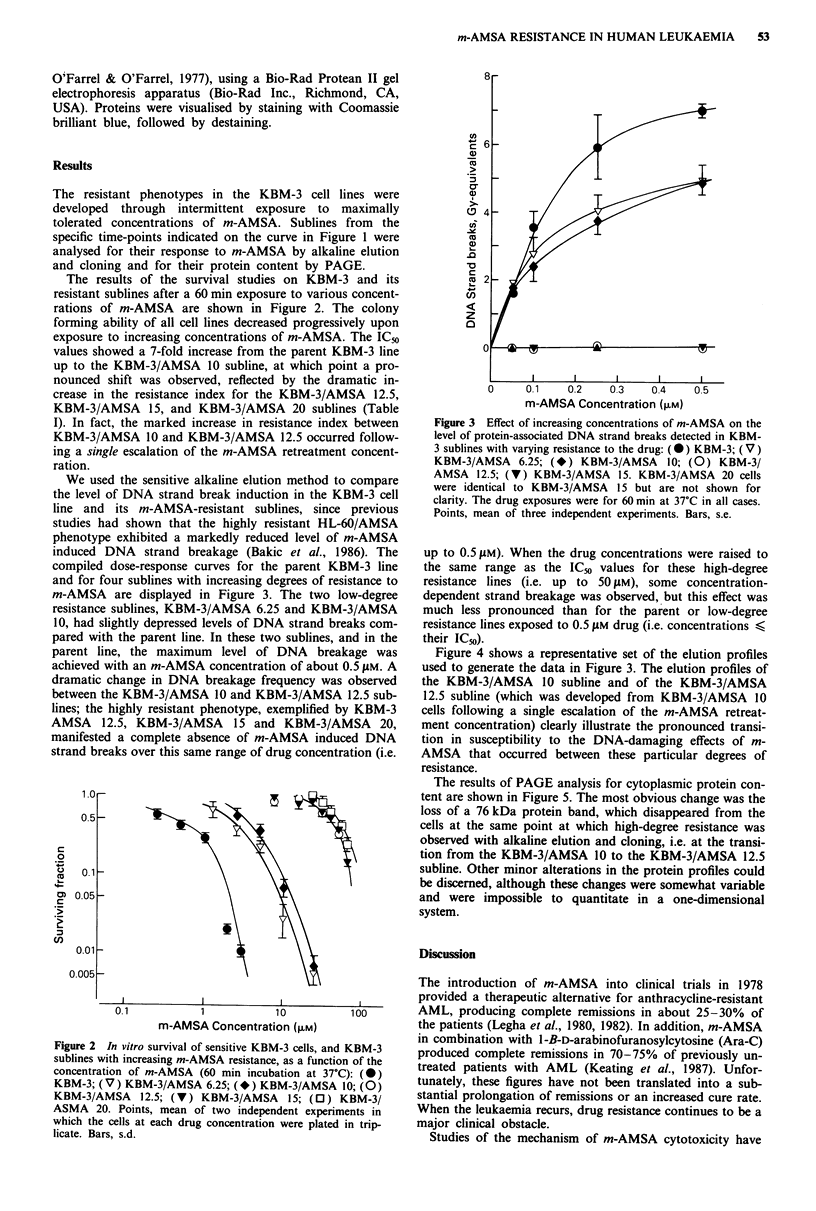

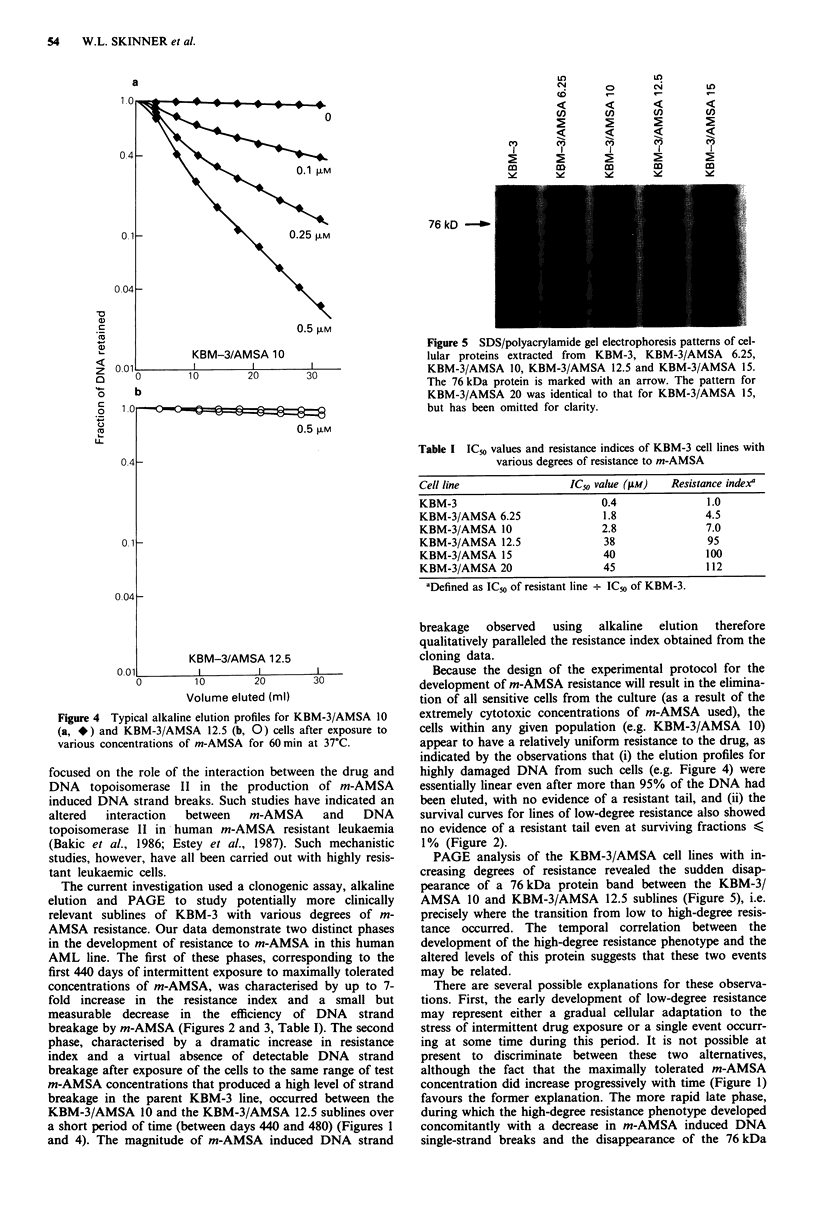

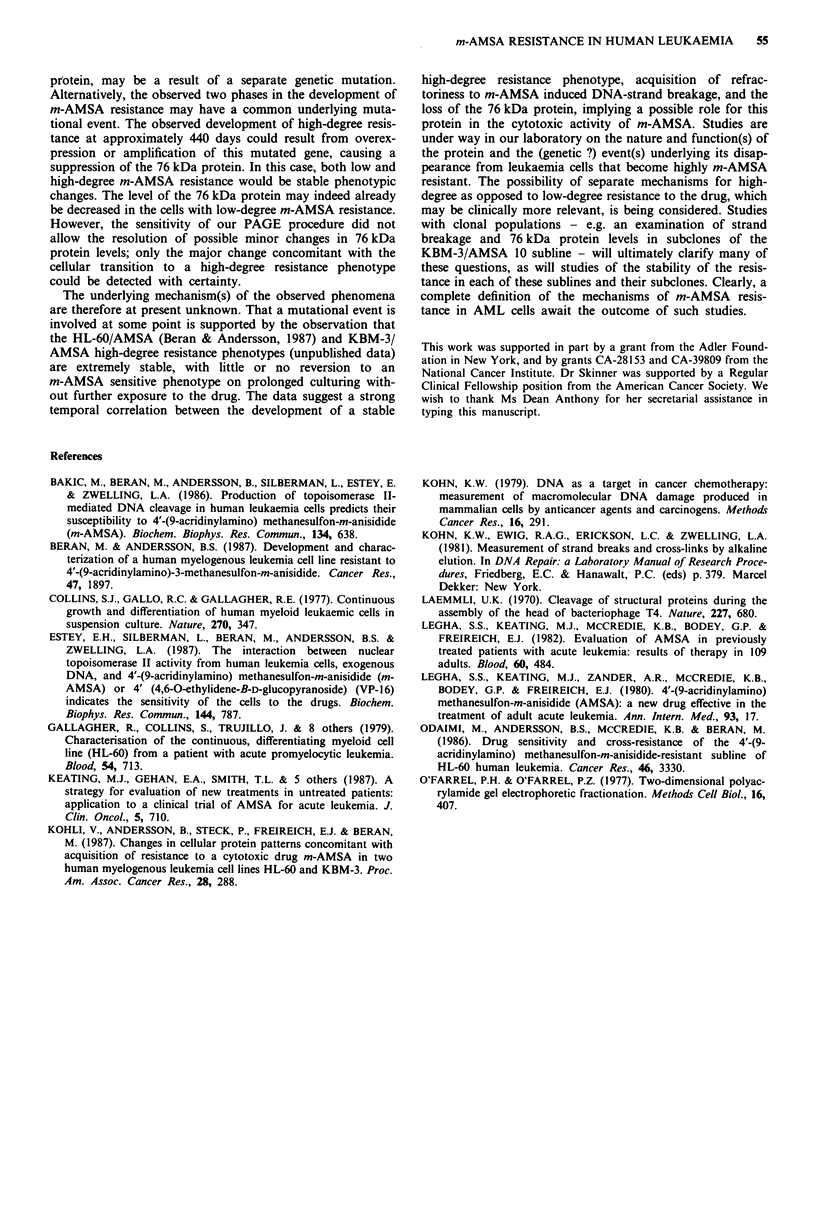


## References

[OCR_00682] Bakic M., Beran M., Andersson B. S., Silberman L., Estey E., Zwelling L. A. (1986). The production of topoisomerase II-mediated DNA cleavage in human leukemia cells predicts their susceptibility to 4'-(9-acridinylamino)methanesulfon-m-anisidide (m-AMSA).. Biochem Biophys Res Commun.

[OCR_00689] Beran M., Andersson B. S. (1987). Development and characterization of a human myelogenous leukemia cell line resistant to 4'-(9-acridinylamino)-3-methanesulfon-m-anisidide.. Cancer Res.

[OCR_00695] Collins S. J., Gallo R. C., Gallagher R. E. (1977). Continuous growth and differentiation of human myeloid leukaemic cells in suspension culture.. Nature.

[OCR_00700] Estey E. H., Silberman L., Beran M., Andersson B. S., Zwelling L. A. (1987). The interaction between nuclear topoisomerase II activity from human leukemia cells, exogenous DNA, and 4'-(9-acridinylamino)methanesulfon-m-anisidide (m-AMSA) or 4-(4,6-O-ethylidene-beta-D-glucopyranoside) (VP-16) indicates the sensitivity of the cells to the drugs.. Biochem Biophys Res Commun.

[OCR_00709] Gallagher R., Collins S., Trujillo J., McCredie K., Ahearn M., Tsai S., Metzgar R., Aulakh G., Ting R., Ruscetti F. (1979). Characterization of the continuous, differentiating myeloid cell line (HL-60) from a patient with acute promyelocytic leukemia.. Blood.

[OCR_00715] Keating M. J., Gehan E. A., Smith T. L., Estey E. H., Walters R. S., Kantarjian H. M., McCredie K. B., Freireich E. J. (1987). A strategy for evaluation of new treatments in untreated patients: application to a clinical trial of AMSA for acute leukemia.. J Clin Oncol.

[OCR_00741] Laemmli U. K. (1970). Cleavage of structural proteins during the assembly of the head of bacteriophage T4.. Nature.

[OCR_00745] Legha S. S., Keating M. J., McCredie K. B., Bodey G. P., Freireich E. J. (1982). Evaluation of AMSA in previously treated patients with acute leukemia: results of therapy in 109 adults.. Blood.

[OCR_00751] Legha S. S., Keating M. J., Zander A. R., McCredie K. B., Bodey G. P., Freireich E. J. (1980). 4'-(9-Acridinylamino) methanesulfon-m-anisidide (AMSA): a new drug effective in the treatment of adult acute leukemia.. Ann Intern Med.

[OCR_00763] O'Farrell P. H., O'Farrell P. Z. (1977). Two-dimensional polyacrylamide gel electrophoretic fractionation.. Methods Cell Biol.

[OCR_00757] Odaimi M., Andersson B. S., McCredie K. B., Beran M. (1986). Drug sensitivity and cross-resistance of the 4'-(9-acridinylamino)methanesulfon-m-anisidide-resistant subline of HL-60 human leukemia.. Cancer Res.

